# The Adaptor Protein SAP Directly Associates with CD3ζ Chain and Regulates T Cell Receptor Signaling

**DOI:** 10.1371/journal.pone.0043200

**Published:** 2012-08-13

**Authors:** Richard Proust, Jacques Bertoglio, Franck Gesbert

**Affiliations:** 1 Institut National de la Santé Et de la Recherche Médicale UMR-S1004, Université Paris-Sud, Hopital Paul Brousse, Villejuif, France; 2 Institut National de la Santé Et de la Recherche Médicale UMR-S749, Institut Gustave Roussy, Villejuif, France; University Paris Sud, France

## Abstract

Mutations altering the gene encoding the SLAM associated protein (SAP) are responsible for the X-linked lymphoproliferative disease or XLP1. Its absence is correlated with a defective NKT cells development, a decrease in B cell functions and a reduced T cells and NK cells cytotoxic activities, thus leading to an immunodeficiency syndrome. SAP is a small 128 amino-acid long protein that is almost exclusively composed of an SH2 domain. It has been shown to interact with the CD150/SLAM family of receptors, and in a non-canonical manner with SH3 containing proteins such as Fyn, βPIX, PKCθ and Nck1. It would thus play the role of a minimal adaptor protein. It has been shown that SAP plays an important function in the activation of T cells through its interaction with the SLAM family of receptors. Therefore SAP defective T cells display a reduced activation of signaling events downstream of the TCR-CD3 complex triggering. In the present work, we evidence that SAP is a direct interactor of the CD3ζ chain. This direct interaction occurs through the first ITAM of CD3ζ, proximal to the membrane. Additionally, we show that, in the context of the TCR-CD3 signaling, an Sh-RNA mediated silencing of SAP is responsible for a decrease of several canonical T cell signaling pathways including Erk, Akt and PLCγ1 and to a reduced induction of IL-2 and IL-4 mRNA. Altogether, we show that SAP plays a central function in the T cell activation processes through a direct association with the CD3 complex.

## Introduction

The signaling lymphocyte activation molecule (SLAM)-associated protein (SAP) is a small cytoplasmic protein encoded by the gene sh2d1a. Mutations or deletions of this gene have recently been shown to be directly responsible for the X-linked lymphoproliferative syndrome-1 (XLP1) [1], [2], [3], [4]. This disease is a rare genetic disorder that only affects young boys and is characterized by an immunodeficiency and an incapacity to mount a proper immune response to Epstein-Barr virus infections [5]. SAP was concomitantly identified as an interactor of the SLAM family of transmembrane molecules. This family of costimulatory receptors includes SLAM (CD150), 2B4 (CD244), NTB-A, CD84, Ly-9 (CD229) and CRACC (CD319) [6], [7], [8], [9]. SAP is a 128 amino-acid long protein and, along with EAT-2 and ERT, it belongs to the SAP family of small adaptor proteins [10]. These small proteins are composed of a single SH2 domain that is followed, in the case of SAP, by a short C-terminal tail. This SH2 domain has been shown to bind to a specific consensus sequence named an Immunoreceptor Tyrosine-based Switch Motif (ITSM), TxYxxV/I/L. This sequence was first evidenced in the cytoplasmic tail of the SLAM family of proteins. It has recently been proposed that SAP plays a switch function. Its recruitment to a specific ITSM may compete with the recruitment of the cytosolic SH2 containing tyrosine phosphatase-2 (SHP2), and may favor the recruitment of SHIP, thus controlling a switch between these two signaling pathways [4], [11].

Also, and this is a unique feature for an SH2 domain, it has been shown that SAP binds several SH3 domain containing proteins, including Fyn, βPix, PKCθ and NCK1 through a region centered on the R78 residue of SAP [12], [13], [14], [15]. This would give SAP the properties of a small adaptor protein, playing a role in the recruitment of signaling molecules to membrane proteins of the SLAM family. Therefore, it is proposed that SAP allows for the recruitment of Fyn to SLAM, and would thus play an activatory role on subsequent signaling mechanisms. The cellular functions of SAP are not yet well understood. SAP is exclusively expressed in T, NK and NKT cells, and its expression in the B cell compartment is still a matter of debate [16], [17]. It has been shown, both in XLP-1 patients and in SAP-deficient mice models, that the absence of SAP is responsible for an immunodeficiency that is due to an absence of NKT cell development, a decrease in B cell functions and a reduced T-cell and NK-cell cytotoxic activities [7], [18], [19], [20].

It is known that CD150 provides a co-stimulatory signal to T cells, and this function is partly dependent on the presence of SAP. T cell activation is dependent upon the triggering of the T cell receptor (TCR)-CD3 complex (TCR-CD3) and the subsequent activation of signaling cascades. The TCR is composed of a heterodimer of α and β variable chains that are responsible for the specific recognition of the antigen properly presented by the MHC molecules [21]. The CD3 complex is composed of a combination of four transmembrane proteins ε, γ, δ and ζ that form hetero- or homodimers (εγ, εδ, ζζ). The unique feature of the T cell antigen receptor is that each CD3 molecule contains at least one ITAM, whose consensus sequence is xYxxI/L(x)_6–8_YxxI/L, in its cytoplasmic tail [22], [23]. Altogether a TCR-CD3 complex contains 10 ITAMs, one for each ε, γ and δ and three for ζ. The triggering of the TCR-CD3 complex leads to a rapid and strong activation of cytosolic tyrosine kinase activity [24], [25], [26], [27]. The Src family kinases Lck and Fyn play a major function in the proximal phosphorylation events mediated by the TCR-CD3 complex. The CD3 molecules are rapidly and sequentially phosphorylated on their ITAM motifs and this leads to an intricate and very organized signaling cascade. The first phosphorylation events that occur on CD3ζ provide a docking site for the tyrosine kinase ZAP70 that, in turn, strengthens the phosphorylation of the chain and also phophorylates accessory molecules [28]. Among these molecules are the transmembrane adaptor protein LAT and the cytosolic adaptor SLP-76 that play central and mandatory functions in the TCR-CD3 signaling events [29], [30]. Once phosphorylated LAT and SLP-76 allow for the subsequent recruitment, directly or indirectly, of signaling molecules such as PLCγ1, PI3K, Cbl, Vav or Grb2 [29], [31], [32], [33].

A full T cell activation requires a signal that is delivered by the TCR-CD3 complex and a co-stimulatory signal from other receptors such as CD28, CD2 and the CD150/SLAM family of receptors. Recent studies have shown that members of this family contain ITSM motifs and thus can associate with SAP and this interaction plays a central role in the co-stimulatory action and regulates some of the TCR-CD3 mediated signaling events [34]. Hence it has been proposed that the XLP associated immune deficiency is due to a lack of T cell activation that would be a consequence of the lack of SAP association with CD150, CD84, NTB-A, CD244 and CD229.

In this paper, we show, in various T cell lines as well as in fresh PBLs that SAP exclusively associates with membrane proteins in activated cells. Among these proteins we have identified NTB-A and more surprisingly CD3ζ. We show that SAP directly binds to residues Y72 and Y83 that compose the first ITAM of CD3ζ (Y72-Y83). Furthermore, we show that SAP deficient cells display a strong reduction of Erk and Akt activation as well as a strong reduction of the recruitment of PLCγ1, Grb2 and SLP76 to a phospho-tyrosine containing complex. Additionally, we show that the silencing of SAP is accompanied by a reduced induction of IL-2 and IL-4 mRNA expression in Jurkat cells. Collectively, these data show for the first time that SAP directly associates with CD3ζ, and this association, in addition to the already described association with SLAM family members, directly regulates TCR-elicited intracellular signaling events and T cell functions.

## Methods

### Reagents and Antibodies

Chemicals were from SIGMA (St Louis, MO) and Euromedex (Souffelweyersheim, France). Restriction enzymes were from New England Biolabs (Ipswich, MA). Fugene reagent was from Roche (Meylan, FR). Anti-CD3ζ and anti-myc (9E10) mAbs, anti-Grb2 and anti-SAP purified sera were purchased from Santa Cruz Biotechnology (TEBU, Le Perray en Yvelines, France), anti-NTB-A mAb from Abnova (Wallnut, CA), anti-phosphotyrosine 4G10 mAb from Upsate Biotechnology (Lake Placid, NY). Monoclonal rabbit Abs against phospho-Akt Ser473 (D9E), phopsho-p44/42 MAPK (Erk1/2, Thr202/Tyr204, D13.14.4E), p44/42 MAPK (Erk1/2, 137F5), phospho-Zap-70 (Tyr319, 65E4), and Zap70 (D1C10E) were purchased from Cell Signaling (Boston, MA). Polyclonal rabbit anti-PLCγ1 and anti-Akt antibodies were from Cell Signaling Technologies (Ozyme, Saint Quentin en Yvelines, France). Anti-beta actin mAb was from MBL (Clinisciences, Nanterre, France), anti-GST antibody was from Neobioscience (Clinisciences) and anti SLP76 mAb was from BD transduction laboratories (BD, Le Pont de Claix, France). For cell stimulation, anti-CD3ε (UCHT-1) mAb was purchased from Biolegend (Ozyme, Saint Quentin en Yvelines, France).

Sh-RNA constructs were from Open Biosystems (Open Biosystems/Thermo Fisher Scientific, Illkirch, France). Two Sh-RNA constructs, targeting different sequences in the SAP transcript, were used in this study. These two constructs, clone ID TRCN0000082710 (Sh-A3) and TRCN0000082712 (Sh-A5), were from the RNAi consortium and there respective sequences are:

5'-CCGGGTGCTGTATCACGGTTACATTCTCGAGAATGTAACCGTGATACAGCACTTTTTG-3′, and 5′-CCGGCACAAGGTACTACAGGGATAACTCGAGTTATCCCTGTAGTACCTTGGTTTTTG-3′.

One Sh-RNAmiR targeting the 3′ UTR sequence of the human CD3ζ transcript was used, ref#V3LHS_409087, mature sens sequence:5′-ACCCGTCAATGTACTAGGATA-3′.

### Cell lines, Culture Conditions and Transfections

Human peripheral blood mononuclear cells were obtained from volunteer anonymous healthy donors from the “Etablissement Français du Sang”. Mononuclear cells were separated by Ficoll-Hypaque gradient centrifugation and treated as described elsewhere [35]. Human samples were collected and handled in the full respect of the declaration of Helsinki.

Jurkat, a human T-ALL derived cell line (ATCC, TIB-152) and H9 (ATCC, HTB-176) a clone derived from the T leukemic cell line Hut-78 were grown in RPMI-1640 medium, supplemented with 10% Fetal Calf Serum (FCS).

Jurkat and H9 cells were transfected using a BioRad (Hercules, CA) electroporator according to the manufacturer specifications. Stable clones were obtained after a 2 week puromycine selection and limiting dilution.

HeLa cell lines (ATCC, CCL-2) were maintained in culture in Dulbecco's Modified Eagle Medium (DMEM, GIBCO/Invitrogen) medium supplemented with 10% fetal calf serum (FCS). Transfections were performed using FuGENE 6 transfection reagent, according to the manufacturer specifications (Roche Diagnostics, Hoffman Laroche, Bazel, Switzerland). The experiments were performed 48 hours post-transfection.

For stimulation experiments, Jurkat and H9 cells were starved over-night in RPMI-1% BSA. The cells were resuspended at 2.10^7^/mL and incubated with either 25 μM pervanadate for 30 min or 10 μg/mL of UCHT-1 Ab for 10 min. Stimulations were stopped with two washes in ice-cold PBS and cells were lysed as described below.

### Plasmids, constructions and site directed mutagenesis

The human CD3ζ was cloned by PCR using the following set of primers 5′-CGGGTACCGCCAC­CATGAAGTGGAAGGCGCTTTTCACC-3′ and 5′-GATATCGCGAGGGGGCAGGGCCTGCATGTG-3′ and inserted into pCR4-TOPO vector (Invitrogen, Carlsbad, CA) according to the manufacturer protocol. For eukaryotic expression, all constructs were subcloned into pEF6-Myc expression vector. CD3ζY-F mutants were generated with the QuickChange® II-E Site-Directed Mutagenesis kit according to the manufacturer instructions (Stratagene Europe, Amsterdam, The Netherlands). The following primers were used for the generation of each mutant, as indicated.

CD3ζ-Y72F: 5′AGAACCAGCTCTTTAACGAGCTCAA3′ and 5′TTGAGCTCGTTAAAGAGCTGGTTCT3′.

CD3ζ-Y83F: 5′GAAGAGAGGAGTTCGATGTTTTGGA3′ and 5′TCCAAAACATCGAACTCCTCTCTTC-3′.

CD3ζ-Y111F: 5′AGGAAGGCCTGTTCAATGAACTGCA3′ and 5′TGCAGTTCATTGAACAGGCCTTCCT3′.

CD3ζ-Y123F: 5′TGGCGGAGGCCTTCAGTGAGATTGG3′ and 5′CCAATCTCACTGAAGGCCTCCGCCA3′.

CD3ζ-Y142F: 5′ACGATGGCCTTTTCCAGGGTCTCAG3′ and 5′CTGAGACCCTGGAAAAGGCCATCGT3′.

CD3ζ-Y153F: 5′CCAAGGACACCTTCGACGCCCTTCA3′ and 5′TGAAGGGCGTCGAAGGTGTCCTTGG3′.

All PCR were performed using high fidelity DNA polymerases and the constructs were verified by DNA sequencing.

### Immunoprecipitation, pull down and Western blotting

Cells were lysed with ice-cold lysis buffer (50 mM Tris (pH 8), 150 mM NaCl, 10 mM NaF, 1 mM EDTA, 1 mM EGTA, and 0,5% Triton X-100) containing 1mM PMSF, 1 mM Na_3_VO_4_ and 1/100 Protease Cocktail Inhibitors. Nuclear pellet and debris were removed by centrifugation at 17×10^3^g 20 min at 4°C. 25 μg of total proteins from cell lysates were mixed with an equal volume of reducing sample buffer 2x (Laemmli's buffer) and boiled for 5 min. For immunoprecipitation experiments, an equivalent amount of proteins from cell lysates was incubated for 2 h at 4°C with the specified antibodies. Protein-G beads were added to the immune complexes for 45 minutes, washed five times with ice-cold lysis buffer. Purified immuno-precipitates, immobilized on protein-G beads, were mixed with an equal volume of Laemmli's buffer 2x and boiled for 5 min.

For pull down experiments, 2−5 μg of purified GST fusion proteins were added to precleared lysates for 2 hours at 4°C. 20 µL of Glutathione-sepharose beads were added for 45 min at 4°C. The beads were washed 5 times with lysis buffer, boiled in Laemmli's buffer.

Proteins were resolved on SDS-polyacrylamide gels and transferred to PVDF membranes. The membranes were blocked for 2 h in TBS-T (TBS/0.2% Tween-20) with 5% BSA prior to being successively probed with the proper primary and secondary Ab in TBS-T. The membranes were washed, and protein detection was conducted using Lycor reagents and Odyssey detection material.

### Far Western Blotting

Proteins were immunoprecipitated, separated, and transferred to PVDF membrane as described above. The membrane was incubated with the indicated purified recombinant protein (GST alone or GST-SAP) at 1 μg/mL in TBS, 0.2% Tween, 3% bovine serum albumin for 1.5 h. The membrane was washed 5 times and the recombinant protein was revealed by western blot against the GST moiety with an anti-GST antibody. The immunoblot was then processed further as described above.

### Cell fractionation

After stimulation, cells were resuspended in Dounce Buffer at 2.10^8^/mL (10 mM Tris (pH 7.6), 0,5 mM MgCl_2_, 1 mM PMSF, 1 mM Na_3_VO_4_ and 1/100 Protease Cocktail Inhibitors) for 10 min at 4°C. Cells were broken by 30 strokes in a Dounce homogenizer with a tight-fitting pestle. Tonicity was restored by adding Tonicity Restoration Buffer (10 mM Tris (pH 7.6), 10 mM MgCl_2_, 1 mM PMSF, 1 mM Na_3_VO_4_ and 1/100 Protease Cocktail Inhibitors). Nuclear fractions were removed by centrifugation at 500 g for 5 min at 4°C. Cytosols and crude membrane fractions were separated by a 45 minutes centrifugation at 110000 g and 4°C in a bench top ultracentrifuge (Optima XL, Beckman- Coulter). The pelleted crude membrane fraction was solubilized in Lysis buffer and the cytosols were equilibrated for the detergent content.

### RNA extraction, Reverse transcription and Quantitative PCR

Cells were incubated on ice for 30 minutes with 10 μg/mL anti-CD3 and 25 μg/mL anti-CD28. After 2 washes in ice-cold PBS, cells were cross-linked with 50 μg/mL goat anti-mouse F(ab')_2_ for the indicated time at 37°C. RNA was extracted using the SV Total Isolation System according to the manufacturer's protocol (Promega). 2 μg of RNA were reverse-transcribed using the SuperScript® III Reverse Transcriptase (Invitrogen). Quantitative PCR were performed in a Stratagene Mx3005P Real Time Thermal Cycler, using the Stratagene Brilliant II SYBR Green QPCR Master Mix (Agilent Technologies) according to the manufacturer's instructions. Transcripts for the ribosomal 60s-subunit protein Rpl38 were used as an internal control. The specific primers used are: Rpl38 forward: 5′-GTTGCTGCTTGCTGTGAGTG-3′; Rpl38 reverse: 5′-CAGATTTGGCATCCTTTCGTC-3′; IL-2, forward: 5′-AACTCACCAGGATGCTCACATTTA-3′, reverse: 5′-TCCCTGGGTCTTAAGTGAAAGTTT-3′; IL-4, forward: 5′-TTCTCCTGATAAACTAATTGCCTCACATTGTC-3′, reverse: 5′-GGTGATATCGCACTTGTGTCCGTGG-3′. Results were expressed as cytokine/Rpl38 mRNA ratio.

## Results

### SAP specifically associates with membrane proteins in activated Jurkat cells

**Figure 1:**
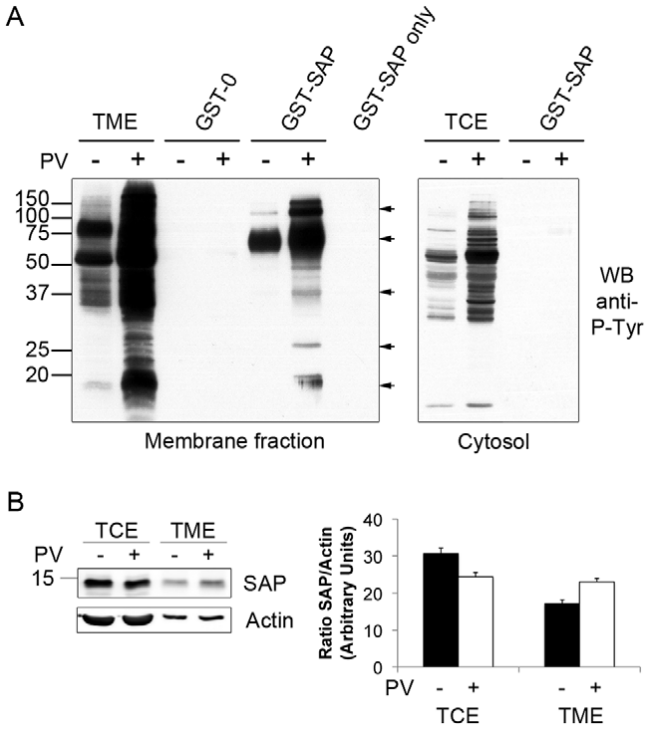
SAP partners are exclusively in the membrane fractions. Jurkat cells were either untreated or treated with 25 µM of pervanadate for 30 minutes. Cells were fractionated by Dounce homogenization and membranes were purified by ultra centrifugation, resolubilized in lysis buffer, precleared and GST or GST-SAP were incubated with the cytosol or membrane fractions. A, pull downs were separated and immunoblotted with anti-phosphotyrosine antibody (4G10). B, crude membrane or cytosolic fractions from 5×10^5^ cells were separated and immunoblotted for SAP. Each band was numerically quantitated (indicated in arbitrary units, a.u.) and the percentages indicate the evolution of the band intensity in the stimulated lysate reported to the non-stimulated lysate in each fraction. These experiments were repeated at least four times. TME: Total Membrane Extract, TCE: Total Cytosolic Extract.

SAP is a small adaptor cytosolic protein that is known to associate with the SLAM family of receptors. In order to further explore the SAP associated signalosome in activated T cells a pervanadate stimulation of Jurkat T cells was performed. It has been described that pervanadate forms a permeant inhibitor of tyrosine phosphatases and leads to an increased tyrosine phosphorylation of many cellular proteins, thus mimicking the pattern of phosphorylation in activated T lymphocytes [36]. The cells were fractionated into a crude membrane fraction and a cytosolic fraction, and equivalent amounts of proteins from each fraction were used for pull down experiments with GST-SAP fusion protein or GST as a control. As shown in figure 1A (left panel), the GST-SAP fusion protein interacts with numerous phospho-proteins extracted from the membrane fraction. The major associated proteins displayed apparent molecular weights spanning from 110 to 21 kDa (figure 1, left panel, arrows). Surprisingly, it did not interact, or only very weakly, with cytosolic proteins (Figure 1A, right panel). Total extracts are shown as Total Membrane extract (TME) and Total Cytosolic Extract (TCE) in Figure 1A.

In addition, a western blot directed against SAP reveals that SAP is slightly more abundant in total membrane extracts (TME) upon activation (Fig 1B, upper panel). The equivalent protein loading, within each fraction, was verified by an anti-actin blot (Figure 1B, lower panel). The intensity of each band was numerically quantified. The densitometric analysis of the SAP/actin ratio, averaged on at least four independent experiments, is represented in Figure 1B. Our results repeatedly showed an increase of SAP in the membrane fraction of activated cells (about 34% increase, in average) with a reciprocal decrease in the cytosolic fraction (about 20% decrease, in average). Altogether, these results indicate that SAP is being recruited at the membrane once its partners are tyrosine phosphorylated. Also these results are the first that demonstrate that SAP exclusively interacts with membrane associated partners and this led us to perform all subsequent experiments with whole cell extracts assuming that all the described interactions occur at the membrane.

### SAP associates with NTB-A and CD3ζ in activated T lymphocytes

**Figure 2:**
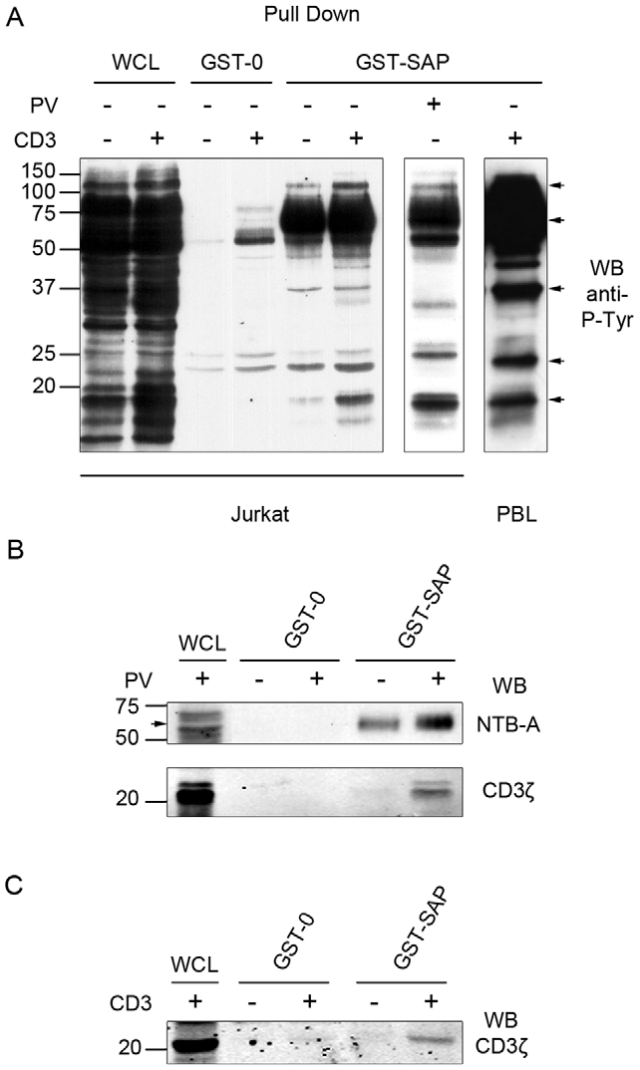
SAP associates with NTB-A and CD3ζ in activated T lymphocytes. *A*, *upper-left panel:* Jurkat cells were stimulated or not with the anti-CD3ε mAb UCHT1 for 10 minutes, lysed, precleared and GST or GST-SAP pull down experiments were performed. *Upper-Middle panel:* Jurkat cells were treated with 25 µM of pervanadate for 30 minutes, lysed, precleared and GST-SAP pull-down was performed as above. *Upper-Right panel:* T-cell blasts were stimulated with UCHT1 for 10 minutes, lysed, precleared and GST-SAP was incubated with cell lysate. Pull downs were immunoblotted with anti-phosphotyrosine (4G10) antibody. *B*, Jurkat cells were either untreated or treated with 25 µM of pervanadate for 30 minutes, lysed, precleared and GST or GST-SAP were incubated with cell lysates. GST pull downs were immunoblotted for NTB-A or CD3ζ, as indicated. *C*, T-cell blasts were stimulated or not with UCHT1 for 10 minutes, lysed, precleared and GST or GST-SAP pull downs were performed and immunoblotted for CD3ζ. These experiments were repeated at least 3 times. The position of NTB-A is indicated by an arrow.

It has been shown that SAP binds to the ITSM sequence located in the cytoplasmic region of the SLAM family of receptors. In order to validate our system we investigated which of these receptors were present in our GST-SAP pull down experiments. GST-pull down experiments were performed on whole cell extracts of resting or CD3-activated cells. This was performed by directly adding a purified UCHT-1 monoclonal antibody, directed against the CD3ε chain. This antibody, used in solution, is known to activate the CD3 complex and the downstream signaling cascade. Noticeably, the pattern of associated proteins, from a whole cell extract, is very similar to the one previously observed in a membrane fraction (compare Fig. 1A to Fig. 2A), thus confirming that the majority, if not all, of the SAP associated proteins is located in the membrane fraction. In more details we could observe several SAP-associated proteins with molecular weights varying from 110, 70, 37, 23 and 20−21 kDa (Fig. 2A arrows). The two stronger signals were obtained with the p70 and the p20−21 phospho-proteins. As a comparison a similar pull down experiment was performed on whole cell lysates from pervanadate-treated Jurkat cells and this produced a very similar pattern of associated phospho-proteins (Figure 2A, compare left and middle panel). This result demonstrates that, in our conditions, a pervanadate activation of T cells perfectly mimics the phospho-signalosome observed with a more physiological TCR-CD3 activation through UCHT1 binding. Also, as shown in figure 2A (right panel) a similar experiment performed on fresh Peripheral Blood lymphocytes provided similar results, thus excluding the possibility that our observation would be cell line specific.

To our knowledge neither SLAM (CD150) nor 2B4, the two main interactors of SAP, are expressed in Jurkat T cells [37]. Using a specific serum (Figure 2B) we show that NTB-A co-migrates with the phosphorylated p70 protein that associates with SAP. Thus NTB-A is at least one component of this heavily tyrosine-phosphorylated p70 band. In accordance with the results obtained in Figure 2A we demonstrate that NTB-A is phosphorylated and associates with SAP in non-activated cells and this association is increased upon activation.

Having confirmed that SAP interacts with a known partner, we turned to the identification of unreported partners. Interestingly, the SAP-associated 20−21 kDa phospho-proteins could be observed in the whole cell lysates of UCHT-1 stimulated cells and were strikingly similar, in their migratory pattern, to the well-known CD3ζ chain. In figure 2B a specific anti-CD3ζ western blot demonstrates that the p20−21 protein is indeed a presumably phosphorylated form of CD3ζ and that it is associated with SAP in activated cells. Additionally, we demonstrate that this SAP-CD3ζ association is also seen in CD3 activated PBL (Figure 2C), which argues against a possible cell line dependent observation.

### SAP directly interacts with CD3ζ

**Figure 3:**
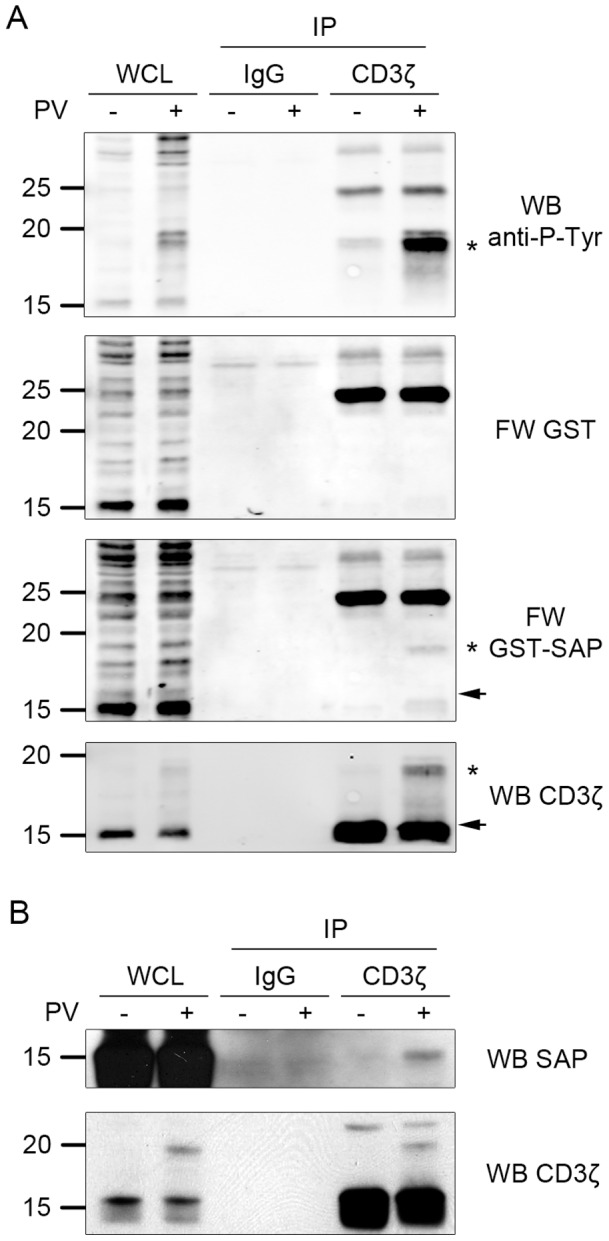
SAP directly associates with phospho-CD3ζ. *A*, Jurkat cells were untreated or treated with 25 µM of pervanadate for 30 minutes, lysed and immunoprecipited with an anti-CD3ζ antibody. The immunoprecipitates were resolved by SDS-PAGE and transferred, the membranes were subjected to Far-western blotting with GST alone (*panel FW GST*) then GST-SAP fusion protein (*panel FW GST-SAP*), stripped and reprobed with an anti-phosphotyrosine (4G10) antibody (*upper panel*) and then with an anti-CD3ζ antibody (*lower panel*). *B*, Jurkat cells were untreated or treated with 25 µM of pervanadate for 30 minutes, lysed, immunoprecipited for CD3ζ and then immunoblotted for SAP (upper panel). The equal amount of precipitated CD3ζ was assessed by an anti CD3ζ blot (lower panel). These experiments were repeated at least 3 times.

The association of SAP with the SLAM family of receptors has been extensively described. In our experiments, we show that NTB-A and CD3ζ precipitate with SAP. The precipitation of CD3ζ in these experiments could be due to an association of the TCR-CD3 complex with NTB-A at the cell membrane, and, thus, CD3ζ could be associated with SAP in an indirect manner. In order to study whether the association of SAP with CD3ζ is direct or indirect, Jurkat T cells were activated with pervanadate and a co-immunoprecipitation assay was performed with an anti-CD3ζ antibody. The membrane was first subjected to a Far-western assay (also called overlay assay), as described in the material and methods section. This method is a method of choice to determine if an association is direct or not. In our case we can demonstrate that SAP directly interacts with CD3ζ p20−21 forms, upon stimulation (stars Figure 3A, panel FW: GST-SAP). These p20−21 forms perfectly overlap the phosphorylated forms observed in an anti-phosphotyrosine immunoblot (stars Figure 3A, panel anti- PTyr). In these experiments we observed that SAP may also associate, under stimulation, with the faster migrating p16 CD3ζ form (arrow Figure 3A, FW GST-SAP and WB CD3ζ panels). In overexposed anti-phosphotyrosine blots we could detect a faint signal at this 16 kDa position, suggesting that the p16 isoform, although at a low level, is also tyrosine phosphorylated (see Figure S1). Noticeably, we did not detect the p16 form in the non-stimulated sample. This point is important as it also rules out any non-specific binding of our fusion protein in the Far-western assay. Also, all the blots were always performed using the same following sequence, FW-GST followed by FW-GST-SAP, followed by immunoblot anti-PTyr and eventually immunoblot anti-CD3ζ. Furthermore, we show in figure 3B that we can indeed co-immunoprecipitate SAP with an anti-CD3ζ antibody, which confirms that the SAP/CD3ζ interaction occurs in vivo and not only in pull down assays. Noticeably, SAP was co-precipitated with CD3ζ only under stimulation, which would preclude an interaction with the non-phosphorylated CD3ζ form. Altogether, these data demonstrate that SAP directly associates with CD3ζ in Jurkat cells.

### SAP-CD3ζ interaction is dependent on the first CD3ζ-ITAM motif

**Figure 4:**
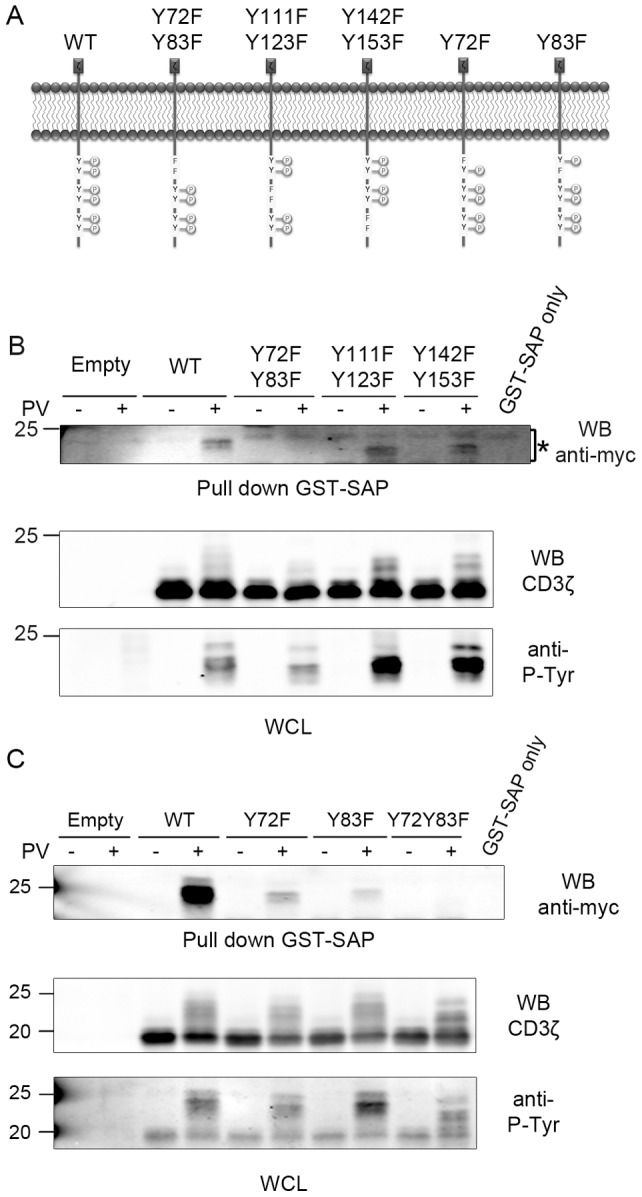
SAP associates with the first CD3ζ-ITAM motif. *A*, Schematic view of the generated CD3ζ mutants. *B*, HeLa cells were transfected with either an empty vector, or with myc-CD3ζ-WT or with mutant myc-CD3ζ as indicated. Forty-eight hours post-transfection the cells were left untreated or were treated with pervanadate for 10 minutes, lysed, precleared and GST-SAP was incubated with the cell lysates. GST-SAP pull downs were immunoblotted with an anti-Myc (9E10) antibody (*upper panel*) and lysates were immunoblotted with anti-CD3ζ and anti-Ptyr antibodies (middle and lower panels, respectively). *C*, HeLa cells were transfected with either an empty vector, or with myc-CD3ζ-WT or with myc-CD3ζ- bearing mutations in the first ITAM, and treated as in *B*. This figure is representative of at least three independent experiments.

As we demonstrated the direct interaction of SAP with CD3ζ, mutants of CD3ζ, in which the cytoplasmic tyrosine residues have been replaced by phenylalanine residues, were generated by site directed mutagenesis and fused to a myc-tag encoding sequence in a eukaryotic expression plasmid. The tyrosine residues were mutated, one by one or ITAM by ITAM, as schematized in figure 4A. In first instance, and in order not to interfere with the endogenously expressed CD3ζ, HeLa cells were transfected with these constructs. Forty-eight hours post transfection the cells were stimulated with pervanadate and a GST-SAP pull down assay was performed. The products of the pull down assays were resolved by SDS-PAGE and detected by an anti-myc immuno-blot (figure 4B, upper panel). As shown, CD3ζ no longer associates with SAP when the first ITAM is mutated (mutant Y72FY83F), whereas the association is not modified when the two other ITAMs are mutated. The apparent molecular weight of CD3ζ in these experiments appears slightly more important than in figures 2 and 3 because of the presence of a C-terminal Myc/6His tag in these constructs. As shown in figure 4B (middle panel) all the constructs were expressed at comparable amounts in the whole cell lysates. In addition, all the constructs were revealed in an anti-phosphotyrosine blot (Figure 4B, Lower panel), excluding the possibility that a lack of interaction would be due to a lack of global phosphorylation of CD3ζ.

As SAP contains only one SH2 domain, we tried to further characterize the exact site of interaction within this proximal ITAM. Wild type, single mutant or double mutant forms of CD3ζ were expressed in HeLa cells treated or not by pervanadate, and the products of a pull down assay were compared (Figure 4C). The equivalent expression of the CD3ζ proteins and their phosphorylation were verified by anti-CD3ζ and anti-phosphotyrosine blots, respectively (Figure 4C middle and lower panels). The GST-SAP precipitated CD3ζ proteins were revealed by an anti-myc immuno-blot (Figure 4C, upper panel). When compared to the WT- CD3ζ, a single tyrosine mutant can still associate with SAP, although to a much lower extent. This suggests that SAP may associate with both tyrosine residues, independently, and that both residues must be mutated in order to completely abrogate the association with SAP.

### Diminished signaling response in T cells in absence of SAP-CD3ζ association

**Figure 5:**
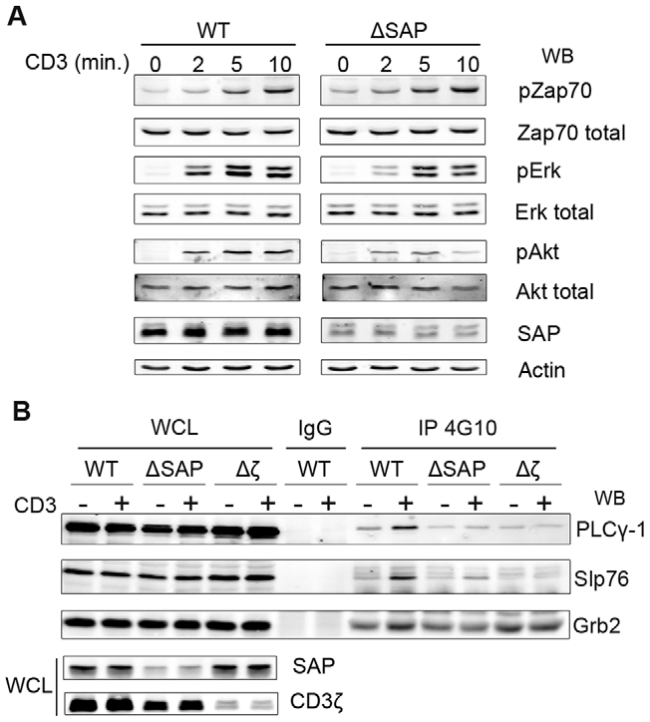
SAP acts in the CD3-mediated activation of Erk, Akt and PLCγ1 pathways. *A*, The parental H9 cell (WT) and H9 cells, stably expressing a SAP specific Sh-RNA (ΔSAP), were stimulated with UCHT-1 for the indicated times. 25 µg of proteins were separated by SDS-PAGE and Zap70, Erk and Akt activations were revealed by Western blotting using specific phospho-antibodies. The total amount of each protein in the lysate was shown for comparison. SAP extinction was controlled by an anti-SAP immunoblotting and equal quantities of proteins in each lane were assessed by an anti-Actin immunoblotting. *B*, parental Jurkat cells (WT) and Jurkat clones expressing either SAP-targeting or CD3ζ-targeting Sh-RNAs (ΔSAP and Δζ respectively) were stimulated or not with UCHT-1 for 10 minutes and lysed. Proteins were immunoprecipited using a phospho-tyrosine antibody (4G10), separated on SDS-PAGE and transferred. PLC-γ1, Slp76 and Grb2 were immunoblotted using specific antibodies. This figure is representative of at least eight experiments in different clones and cell lines.

As we have evidenced a direct association of SAP with CD3ζ in activated T lymphocytes, we investigated whether we could show an effect of the absence of SAP on the proximal CD3 mediated signaling events. For that purpose we generated H9 cell lines deficient for SAP expression. This was performed through the constitutive expression of Sh-RNA designed to specifically down-modulate the expression of SAP. The cells were selected with the appropriate selection agent and a polyclonal population, that displayed an average 80% down-modulation of SAP expression, was used in our experiments (figure 5A, SAP panel, compare left and right panels). Similar results were obtained with two different Sh-RNA constructs, as specified in the material and methods section. The results shown in figure 5 were obtained with the SAP Sh-A3 and were confirmed with the SAP Sh-A5 (not shown). In order to study proximal signaling events, the cells were stimulated with UCHT-1 for the indicated period of time. At first, we could observe that ZAP 70 phosphorylation, evidenced by a phospho specific antibody, was not modified in cells that do not express SAP (Figure 5A, pZAP70 panel).

Further investigations showed that ΔSAP H9 cells displayed a noticeable and reproducible reduction of both Erk and Akt phosphorylation (figure 5A, pErk and pAkt panels, respectively, compare left and right panels). Similar results were obtained in Sh-SAP expressing Jurkat cells (Not shown). However, as H9 cells are known to express both SHIP and PTEN, which is not the case of Jurkat cells, the reduced phosphorylation of Akt was easier to evidence in H9 cells [38]. Similar stimulation assays were performed in the parental Jurkat cell line and in clones that have an Sh-RNA-mediated lower expression level of SAP or CD3ζ (ΔSAP or Δζ respectively). After stimulation, the cells were lysed and subjected to an anti-phosphotyrosine immunoprecipitation. The products were resolved by SDS-PAGE and revealed by immunoblotting against various signaling molecules known to precipitate or co precipitate in these conditions. In figure 5B we can show that the wild type parental cell line responded nicely to the CD3 stimulation and we could evidence, as expected, a good co precipitation of three major CD3-mediated signaling molecules, namely SLP76, Grb2 and PLCγ1. As expected, in the cells that do not express CD3ζ (Δζ), we could barely detect SLP76 and PLCγ1 in 4G10 IPs. On the contrary, and for reasons that remain unclear, the level of phosphotyrosine associated-Grb2 was constitutively high and did not respond to the stimulation by UCHT1. Interestingly, the Jurkat clones that have a reduced expression level of SAP (ΔSAP) displayed an intermediary pattern, with an important reduction, although not complete, of the 4G10-associated SLP76. More strikingly, the levels of Grb2 and PLCγ1 were constitutively low and did not increase in response to CD3 stimulation.

**Figure 6:**
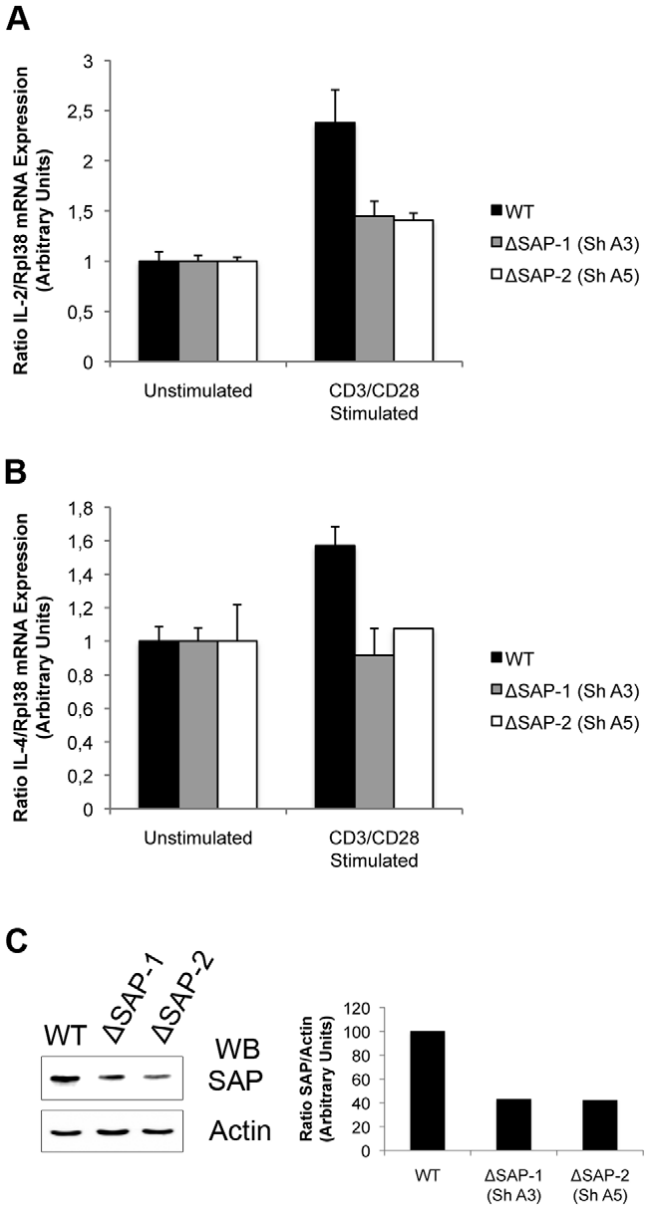
SAP-silencing down-modulates IL-2 and IL-4 gene expression in Jurkat cells. *A and B*, The Jurkat parental cell (WT) and two clones (ΔSAP-1 and ΔSAP-2), stably expressing two different SAP specific Sh-RNAs (Sh-A3 and Sh-A5 respectively), were stimulated by CD3/CD28 for 60 min for IL-2 (A) or 30 min for IL-4 (B). Quantitative RT-PCR was performed for IL-2 (A) and IL-4 (B) gene expression. Results were normalized to the ribosomal protein, Rpl38, gene expression. Data shown are representative of two independent experiments. *C*, Whole cell lysates of the Jurkat parental cell (WT) and the two clones ΔSAP-1 and ΔSAP-2 were resolved by SDS-PAGE and SAP extinction was controlled by an anti-SAP immunoblotting. Equal amount of proteins in each lane were assessed by an actin-immunoblotting. Each band was numerically quantitated (indicated in arbitrary units, a.u.) and the SAP/Actin ratio for each cell population is graphically presented (normalized to 100% in Jurkat WT).

As we can evidence a decrease in some signaling events downstream of a TCR-triggering in SAP deficient cells, we seeked for a more functional relevance of SAP involvement in this particular cascade. The production of IL-2 and IL-4 is a known functional consequence of the antigen-stimulation of T cells that would eventually lead to Th_2_ differenciation [39]. Both cytokines have been shown to be expressed by CD3/CD28-stimulated jurkat cells [40]. We used a quantitative PCR method in order to quantitate a possible modulation of IL-2 and IL-4 gene expression in ΔSAP cells compared to wild-type jurkat cells. The cells were either left untreated or were CD3/CD28-stimulated. RNA was extracted and amplified as described in the material and methods section. In Figure 6, we show that in two different populations of jurkat cells, independently silenced with two different SAP-targeting Sh-RNAs (Sh-A3 and Sh-A5), the down-modulation of SAP is accompanied by a reduction of the induction of both IL-2 and IL-4 gene expression in response to TCR stimulation. As represented in Figure 6A and 6B, we observed an overall 38% decrease of the induction of IL-2 and IL-4 gene expression, respectively, when the SAP expression is down modulated by about 58% (as represented in Figure 6B).

Altogether our results show that SAP does not modify the activation of a very proximal event such as ZAP70 phosphorylation. However, it plays a role in the activation of canonical signaling pathways such as PLCγ1, Grb2, Erk and Akt. Furthermore, the absence of SAP expression correlates with a lack of induction of more distal and functional events such as the gene expression of IL-2 and IL-4.

## Discussion

In this study we document a novel direct interactor of the CD3ζ chain, namely the SH2 domain-only molecule SAP. Despite its single SH2 domain structure, SAP has been shown to interact with numerous proteins that mostly belong to the SLAM family of receptors [6], [9]. Others have extensively studied the interaction of SAP with the SLAM-CD150 family of proteins.

A full T cell activation requires a signal that is delivered by the TCR-CD3 complex and a co-stimulatory signal from other receptors such as CD28. In addition to CD28, receptors belonging to the ITSM-containing CD150 (SLAM) family have been demonstrated to mediate a co-stimulatory signal. Recent studies have shown that members of this family contain ITSM motifs and thus can associate with SAP and this interaction plays a central role in the co-stimulatory action and regulates some of the TCR-CD3 mediated signaling events. Hence it has been proposed that the XLP associated immune deficiency is due to a lack of T cell activation that would be a consequence of the lack of SAP association with CD150, CD84, NTB-A, CD244 and CD229.

Data obtained by others, in T cells from XLP patients, have suggested that SAP may play a direct role in the TCR-CD3 mediated signal [34]. Through a non-elucidated mechanism, these authors demonstrate that a default of SAP expression is accompanied by a default of activation of some canonical signaling pathways, such as Erk and Vav phosphorylation.

Our work provides a mechanism for the function of SAP in the TCR-CD3 activatory signaling. In the present paper, we show that, in activated T cells, SAP interacts with NTB-A, a CD150 (SLAM) family member, as well as with the CD3ζ chain. We demonstrate that the CD3ζ-SAP interaction requires the first ITAM motif in the cytoplasmic tail of CD3ζ. Furthermore, by the mean of Far Western blotting, we show that this interaction is direct. Additionaly, we can exclude the possibility that the interaction between SAP and CD3ζ would be mediated, in co-immunoprecipitation experiments, through NTB-A. Indeed, we can evidence this interaction in HeLa and Kit-225 cell lines that do not express NTB-A. Altogether, these observations confirm that the SAP-CD3ζ association described in this paper is not mediated by NTB-A.

To our knowledge, this is the first demonstration that SAP interacts with CD3ζ, directly, and that by such an association it directly plays a function in the TCR-CD3 mediated signaling (i.e. not through SLAM receptors). We demonstrate that, when the expression of SAP is down modulated, the phosphorylation of Erk and of Akt is diminished. These results suggest that the function of SAP is to favor the activation of the Erk and Akt pathways downstream of ZAP70 activation. In agreement with our results, and while this manuscript was in revision, Baldanzi et al, with one of the Sh-RNA construct used in the present study (clone ID TRCN0000082712), showed that the down-modulation of SAP in Jurkat cells leads to a decreased Erk activation in response to a CD3-CD28 stimulation [41]. We show that in absence of SAP the PLCγ1 protein is not recruited anymore to phosphorylated complexes. This observation could confirm that SAP may inactivate DGKα through the activation of PLCγ1, as suggested by Baldanzi et al.

Whether SAP functions downstream of ZAP70 or independently of ZAP70 is still a matter that we were not able to address. Altogether our data partly confirm the results obtained by Nakamura *et al*. in HVS-transformed cells from XLP patients [42]. However, these authors described an increase of CD3ζ and ZAP70 phosphorylation. These discrepancies might be explained by the facts that the patient cells used in their studies were virally transformed and probably in a constitutively activated state. Interestingly the p75 SAP-associated protein described by Nakamura et al. is most probably the NTB-A protein that we identified in the present work.

We provide evidence that in absence of SAP the recruitment of PLCγ1, Grb2 and SLP76 to phosphotyrosine containing complexes is largely impaired. Additionally we show that the silencing of SAP expression leads to a noticeable down-modulation of IL-2 and IL-4 gene induction, as assessed by quantitative PCR. Altogether, our results demonstrate that SAP directly plays a central function in T cell activation. We have shown that in absence of SAP, the global phosphorylation of ZAP70 remains unchanged when compared to non-modified cells (Figure 5). It would then be of great interest to determine which proximal event is affected by the absence of SAP in the CD3 mediated signaling events.

Also we performed similar experiments in CD3ζ depleted Jurkat cells re-expressing the various ITAM mutants as described in figure 4 (data not shown). In these cells we did not evidence any dramatic modification of the studied signaling events. This observation would suggest that, in agreement with Malissen *et al*., when CD3ζ ITAM motifs are crippled some other CD3 components (i.e. CD3ε or CD3γ) may participate in the activation events [43]. This could also be true for SAP, and it may be recruited to another component when CD3ζ is not functional.

The SAP protein has been demonstrated to bind to a consensus sequence T/SxYxxV/I/L, named ITSM that is present in all the SLAM family of receptors [44]. We have identified the site of interaction of SAP on the first ITAM motif of CD3ζ. This is a strict consensus sequence for an ITAM motif, and it does not present the canonical structure of an ITSM, with a T or S in “−2” from the Y residue.

Thus we provide evidence that, *in-vivo*; SAP may also bind to structures other than ITSM motifs. Li et al showed that the SAP SH2 domain is very flexible and that it adopts a different structure according to the sequence of the peptide it complexes with. Furthermore, Li et al showed that SAP displays various relative affinities depending on the type of interaction it can make with its target motifs [45]. Accordingly, they evidenced that the lowest relative affinity is obtained between the SAP-SH2 domain and a conventional “two-pronged” YxxV/I consensus motif. Thus, our results confirm that SAP may behave as a conventional SH2 domain bearing protein. However, the fact that this interaction may be of a much lower affinity may explain why it has not been described earlier and was probably masked by the association with SLAM family receptors. Additionally, we have shown in a Far-Western assay that SAP almost exclusively binds directly and in an inducible manner to the phosphorylated p21 form of CD3ζ (see figure 3A, stars). These various phosphorylated forms of CD3ζ have been described in the past and are regularly observed [46]. However, we could also detect a slight association of SAP with a p16 form of CD3ζ, under stimulation. We have observed that this form is slightly phosphorylated (see Figure S1) and also we may favor such an interaction in vitro on a denatured CD3ζ. In conclusion, we show that SAP interacts in an inducible manner with the phosphorylated CD3ζ chain, this could make the difference between an ITSM and an ITAM motif, and only an ITSM motif would allow a constitutive interaction with SAP whereas an ITAM needs to be phosphorylated. Furthermore, all the three ITAMs contained in the cytoplasmic tail of CD3ζ present a similar consensus sequence. It is thus tempting to speculate that either a conformational event or the occupation of the other ITAM motifs by other signaling molecules renders only the first ITAM accessible for SAP binding. However this observation broaden the possible function of ITAM motifs, as it has been suggested they may already overlap the functions of ITIM motifs thus being called inhibitory ITAMs [47], [48].

Our study confirms that SAP plays a major role in the regulation of signaling events that control the activation of T lymphocytes. Of note, the results presented here may highlight only a partial image of the function played by SAP in the TCR signaling. SAP has been shown to associate with other partners, through a R78/SH3 interaction, as it does with Fyn. Among these partners are PKCθ and βPix that have been shown to play important function in T cell signaling. It is indeed tempting to speculate that the function of SAP would be to bring additional partners, such as Fyn or PKCθ, in proximity of the CD3 complex. An association of SAP with Fyn could be confirmed in our investigations (not shown) and it would probably be of interest to further decipher the signalosome that is brought to the TCR through its direct interaction with SAP. It is interesting to note that, in our cellular model, SAP associates with NTB-A and CD3ζ and seems to participate in proliferation and survival mechanisms. It has been proposed in the past that IL-2 signaling could propagate signals that uncouple the TCR from the CD3 complex and therefore modify the ability of the cells to respond through the full TCR/CD3 complex [49]. It is thus possible that in response to some stimuli (i.e. IL-2), these interactions would be modified and the cells would respond differently to a re-stimulation of their TCR complex leading to an apoptotic signal as recently reported by Snow et al [50].

While this manuscript was in preparation Bida et al showed that 2B4 (a member of the CD150-SLAM family) uses ITAM containing molecules, such as CD3ζ, to initiate signaling [51]. However, through co-immunoprecipitation experiments they did not demonstrate that SAP directly interacts with CD3ζ, and it could not be excluded that the SAP-CD3ζ occurs through the interaction with a common partner. Also, they showed that SAP could be constitutively co-precipitated with CD3ζ in NK cells. These results are in agreement with our observation, however it seems that this interaction is more inducible in T cells. The observation by Bida *et al*. reinforces the idea that SAP, through its interaction with CD3ζ, may play a more general role in the activation of cells using CD3ζ-ITAMs. The association and the functions of SAP that are shown in the present study would be worth being investigated in other cell types using CD3ζ-ITAM signaling such as NK or B cells expressing CD16-CD3ζ complexes.

Our data suggest that SAP plays a complex role that cannot exclusively be related to its association with the SLAM family members. Supporting this idea are the recently published data by Li *et al*. [52] that showed that SAP associates with FCγRIIB in B-lymphocytes and by recruiting Lyn kinase plays an inhibitory function in B cell signaling. It is also possible that this interaction of SAP with CD3ζ may be crucial for the development and function of NKT cells that have been shown to be absent in XLP1 patients or in SAP deficient mice.

## Supporting Information

Figure S1 **CD3ζ migrates as several phosphorylated bands upon pervanadate treatment.** Jurkat cells were left untreated or were treated with pervanadate for 30 minutes. Cells were lysed and immunoprecipitates were performed, washed and resolved as described in the material and methods section. After transfer, the membrane was first immunoblotted with an anti-PTyr antibody (upper panel). The presence of CD3ζ was checked by an anti-CD3ζ immunoblot (lower panel). Arrows indicate all the CD3ζ forms.
(TIF)
