# Supplementary figures and images for: The Adaptor Protein SAP Directly Associates with CD3ζ Chain and Regulates T Cell Receptor Signaling
Source: PLoS One. 2012 Aug 13;7(8):e43200. doi: 10.1371/journal.pone.0043200 (PMC3418226; doi:10.1371/journal.pone.0043200)

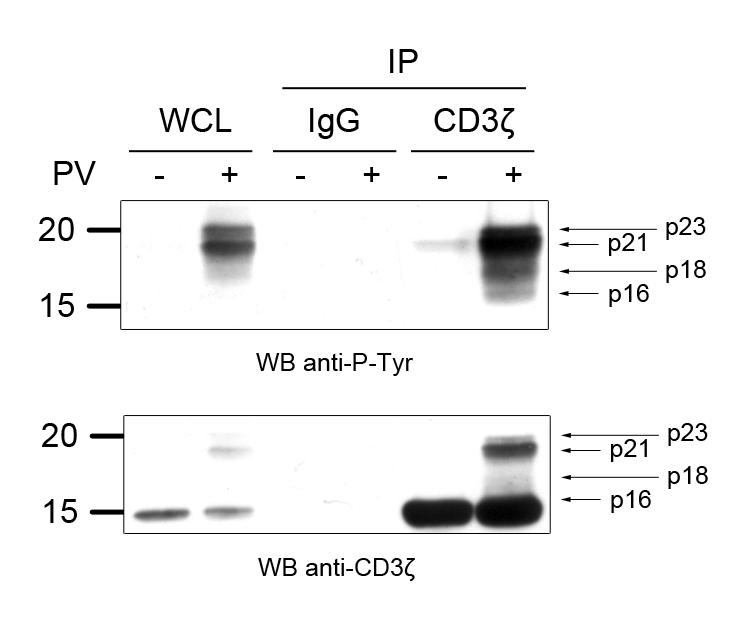

Supplement: Figure S1 — CD3ζ migrates as several phosphorylated bands upon pervanadate treatment. Jurkat cells were left untreated or were treated with pervanadate for 30 minutes. Cells were lysed and immunoprecipitates were performed, washed and resolved as described in the material and methods section. After transfer, the membrane was first immunoblotted with an anti-PTyr antibody (upper panel). The presence of CD3ζ was checked by an anti-CD3ζ immunoblot (lower panel). Arrows indicate all the CD3ζ forms. (TIF) [file pone.0043200.s001.tif]
